# Characterization of the Major Odorants in Non-Alcoholic Beers Brewed with Either *Kluyveromyces marxianus* or *Cyberlindnera saturnus*

**DOI:** 10.3390/foods15173029

**Published:** 2026-08-27

**Authors:** Stephanie Frank, Robin Maier, Martin Steinhaus

**Affiliations:** Leibniz Institute for Food Systems Biology at the Technical University of Munich (Leibniz-LSB@TUM), Lise-Meitner-Straße 34, 85354 Freising, Germany; s.frank.leibniz-lsb@tum.de (S.F.);

**Keywords:** non-*Saccharomyces* yeast, *Kluyveromyces marxianus*, *Cyberlindnera saturnus*, non-alcoholic beer, gas chromatography–olfactometry (GC–O), aroma extract dilution analysis (AEDA), stable isotopically substituted odorants, odor activity value (OAV), 3-methylbutyl acetate

## Abstract

Consumer behavior is increasingly shifting toward non-alcoholic beers. However, dealcoholized beers often have a reduced aroma compared to their alcoholic counterparts. An alternative for producing non-alcoholic beers is the use of special yeasts with reduced sugar metabolization; however, there are still significant gaps in our knowledge regarding the molecular contribution of non-*Saccharomyces* yeasts to beer aroma. Applying a comparative aroma extract dilution analysis to two non-alcoholic beers brewed with *Kluyveromyces marxianus* or *Cyberlindnera saturnus* resulted in 31 odor-active compounds, among which 27 were detected in both beer samples. Sensory evaluation showed well-balanced aromas in the two beers, with a more intense banana note in the beer brewed with *C. saturnus*. Among the 22 odorants that exceeded their odor threshold concentrations in the *C. saturnus* beer was the banana-like smelling 3-methylbutyl acetate, which showed a significantly lower odor activity value in the *K. marxianus* beer (OAVs 830 vs. 8.2). Its decisive role in the banana note of the *C. saturnus* beer was finally confirmed in a spiking experiment.

## 1. Introduction

In recent years, a change in the population’s drinking behavior has been observed. The consumption of non-alcoholic beer is steadily increasing. Per capita sales in Germany rose from 2.95 L in 2014 to 4.99 L in 2024, and the forecast for the next few years suggests a further increase to 5.55 L in 2027 [1]. Greater health awareness is a key driver of this trend. However, non-alcoholic beers frequently have a weaker aroma compared to their alcoholic counterparts and sometimes even exhibit off-flavors. Without this quality impairment, consumer acceptance of non-alcoholic beers could be even higher.

According to Regulation (EU) No. 1169/2011 of the European Parliament and of the Council, the ethanol content of beverages within the European Union must be indicated when it exceeds 1.2% ALC/VOL. The situation is different when it comes to the definition of the term “non-alcoholic”. There are no uniform regulations throughout the European Union. In most EU countries, including Germany, beverages containing no more than 0.5% ALC/VOL are labeled as non-alcoholic. This paper will follow this terminology.

Non-alcoholic beer is generally produced in two different ways. Either the ethanol is removed from the finished beer after the conventional brewing process, or the yeast’s ethanol formation during fermentation is limited. Methods based on the former principle are called physical methods, and those based on the latter are called biological methods [2]. Physical methods remove the ethanol through thermal processes (e.g., evaporation and rectification) or membrane processes (e.g., dialysis, reverse osmosis, osmotic distillation, and nanofiltration) [2,3,4]. Biological methods limit ethanol production by reducing fermentable sugar content, altering fermentation conditions to reduce yeast activity, or using selected yeasts with low fermentation ability [2,3,4]. Investigations into the application of non-*Saccharomyces* yeasts have gained momentum in recent years [3]. Numerous non-*Saccharomyces* yeast species that produce low- or non-alcoholic beers have been identified [5,6,7,8,9,10]. Two of these are *Kluyveromyces marxianus* and *Cyberlindnera saturnus*, both of which are maltose-negative. Previous studies on the aroma of beers brewed with *K. marxianus* [5,11,12,13,14,15] or *C. saturnus* [5,13,16,17] were focused on the identification of volatile compounds; however, the aroma relevance of the individual compounds was rarely assessed [5,13,17]. We recently conducted a systematic gas chromatography–olfactometry (GC–O)-based study on the aroma of an alcoholic beer brewed with *Torulaspora delbrueckii* and successfully identified ethyl butanoate, ethyl 2-methylpropanoate, and ethyl propanoate as the compounds responsible for the beer’s characteristic fruity aroma [18]. Sensory verification of analytical data is essential to establish causal relationships between sensory and analytical data and avoid false conclusions from correlations. However, such studies on the aroma of non-alcoholic beers brewed with either *K. marxianus* or *C. saturnus* have been lacking. The aims of the current study were (1) to identify the major odor-active compounds in non-alcoholic beers brewed with either *K. marxianus* or *C. saturnus* by combining GC–O screening and aroma extract dilution analysis (AEDA); (2) to quantitate potent odorants in the two beer samples and calculate odor activity values (OAVs); and (3) to verify the data by sensory experiments.

## 2. Materials and Methods

### 2.1. Beers

Two experimental beers were produced at the Research Center Weihenstephan for Brewing and Food Quality (BLQ; Freising, Germany) using 30 L batches of wort. Fermentation was carried out with either *Kluyveromyces marxianus* strain TUM G4KM (Km4) or *Cyberlindnera saturnus* strain YHMH22AA-3H1 (CSa1). The wort was made from malt extract (Weyermann Specialty Malting; Bamberg, Germany) and did not contain hops. Its original gravity was standardized to 7 °P, and yeast was pitched at a concentration of 15 × 10^6^ cells/mL. Fermentations were conducted at 20 °C for 6 days, followed by a cold-conditioning period at 2 °C for 96 h. The finished beers were subsequently bottled and pasteurized at 80 PE. The final ethanol content was determined using the MEBAK (Central European Commission for Brewing Analysis) standard method [19] and was 0.4% ALC/VOL (Km4 beer) and 0.5% ALC/VOL (CSa1 beer).

### 2.2. Chemicals

Reference odorants **1**–**7**, **8a**, **8b**, **9**, **11**–**27**, and **29**–**38** were purchased from Merck (Darmstadt, Germany). Compound **10** was prepared following the synthetic procedure reported previously in the literature [20]. The following stable isotopically substituted odorants were acquired from commercial suppliers: (^2^H_10–11_)-**9**, (^2^H_3_)-**11**, (^2^H_6–8_)-**24**, (^13^C_2_)-**30**, and (^13^C_2_)-**36** (Merck); (^2^H_7_)-**13** and (^2^H_3_)-**18** (Cambridge Isotope Laboratories; Tewksbury, MA, USA); (^2^H_5_)-**20** and (^2^H_2_)-**33** (CDN Isotopes; Pointe-Claire, QC, Canada); and (^13^C_2_)-**22** (aromaLAB; Planegg, Germany). The following stable isotopically substituted odorants were synthesized according to published methods: (^2^H_3_)-**1** [21], (^2^H_5_)-**2** [22], (^2^H_3_)-**3** [23], (^2^H_3_)-**5** [24], (^2^H_11_)-**7** [22], (^2^H_2_)-**8b** [25], (^13^C_5_)-**10** [20], (^2^H_3_)-**12** [23], (^2^H_2_)-**14** [22], (^2^H_2_)-**15** [24], (^2^H_3_)-**16** [26], (^2^H_4–7_)-**17** [27], (^13^C_2_)-**21** [28], (^13^C_2_)-**25** [29], (^2^H_3_)-**26** [30], (^2^H_3_)-**27** [31], (^2^H_3_)-**31** [30], (^2^H_2_)-**34** [32], (^13^C_4_)-**35** [33], (^2^H_2_)-**37** [32], and (^2^H_2_)-**38** [32]. Diethyl ether was sourced from VWR (Darmstadt, Germany) and was freshly distilled prior to its application.

### 2.3. Gas Chromatography (GC)

GC analyses were accomplished using a GC–O/FID instrument, a one-dimensional HS–GC–O/MS instrument, a one-dimensional GC–MS instrument, a comprehensive two-dimensional GC×GC–MS instrument, or a two-dimensional heart-cut GC–GC–MS instrument. Details of the GC instruments and the applied parameters are available in the Supplementary Materials file and our previous paper [18]. During GC–O analysis, the column effluent was assessed by a trained and experienced assessor positioned at the sniffing port. Odor events were documented by noting both their retention times and perceived odor qualities whenever they occurred. To ensure reliable odor detection, all olfactometric analyses were performed independently by three assessors possessing complementary olfactory capabilities [34]. Retention indices (RIs) were determined from GC measurements performed under a linear oven temperature program of 6 °C/min. RIs were calculated by interpolating the retention time of each analyte between those of the neighboring *n*-alkanes [35].

### 2.4. AEDA

The isolation of volatiles is described in detail in our previous paper [18]. In brief, after decarbonization, beer (250 g) was vigorously shaken with diethyl ether, nonvolatiles were removed by solvent-assisted flavor evaporation (SAFE) [36] at 40 °C, and the volatile fraction was concentrated to 1 mL using a Vigreux column (50 × 1 cm) and a Bemelmans microdistillation device [37]. The concentrated volatile isolates of the two beer samples were serially diluted 1:2 with diethyl ether. The original samples as well as each diluted sample were analyzed by GC–O using the GC–O/FID instrument with a DB-FFAP column. For every odor-active compound, a flavor dilution (FD) factor was established as the highest dilution at which the corresponding odorant quality could still be perceived at the sniffing port by at least one of the three trained assessors during GC–O evaluation.

### 2.5. Headspace Dilution Analysis

An aliquot of beer (20 g) was placed into a headspace vial (120 mL) sealed with a septum and agitated for 30 min at ambient temperature (22 ± 1 °C). Subsequently, headspace samples of varying volumes (20, 10, 5, 2.5, and 1.25 mL) were withdrawn using a gas-tight syringe and introduced into the HS–GC–O/MS instrument equipped with a DB-5 column. For each odor-active compound, an FD factor was determined as the quotient of the largest injected headspace volume (20 mL) and the smallest volume at which the odorant remained perceptible at the sniffing port to any of the three assessors during HS–GC–O analysis.

### 2.6. Odorant Quantitation

Quantitative analysis of the compounds **2**, **3**, **7**–**22**, **24**–**27**, **30**, and **31** was performed by adding diethyl ether (10–200 mL) together with stable isotopically substituted odorants (0.05–1 µg) to degassed beer samples (1–150 g). The resulting mixtures were agitated for 30 min at ambient temperature (22 ± 1 °C). Volatiles were subsequently isolated according to the procedure reported in our previous paper [18]. The volatile isolates were concentrated to volumes between 100 µL and 1 mL. Depending on the analyte, the final concentrates were analyzed either by the one-dimensional GC–MS instrument (**11**, **13**–**15**, and **18**) or the comprehensive two-dimensional GC×GC–MS instrument (**2**, **3**, **7**–**10**, **12**, **16**, **17**, **19**–**22**, **24**–**27**, **30**, and **31**). For the determination of the compounds **1**, **4**, **5**, **33**–**37**, and **38**, an aliquot of beer (20 g) was placed into a headspace vial (120 mL) sealed with a septum. Stable isotopically substituted odorants (0.1–1 µg) were added as internal standards, and the mixture was agitated for 30 min at ambient temperature (22 ± 1 °C). Finally, a 20 mL aliquot of the headspace was analyzed using the one-dimensional HS–GC–O/MS instrument.

Peak areas of the analytes and the corresponding internal standards were obtained from extracted ion chromatograms using the quantifier ions listed in the Supplementary Materials file, Table S1. Concentrations of the target compounds in the beer samples were calculated from the area counts of the analyte peaks, the area counts of the standard peaks, the amounts of beer sample used, and the amounts of internal standard added using the respective calibration line equation provided in the Supplementary Materials file, Table S1. The calibration line equations were obtained by analyzing mixtures with different analyte-to-standard concentration ratios under identical conditions, followed by linear regression.

Compound **39** was quantitated using an enzymatic assay based on a commercially available kit supplied by Megazyme (Bray, Ireland).

### 2.7. Quantitative Olfactory Profile Analyses and Spiking Experiment

The sensory panel consisted of 20 trained participants (8 males and 12 females) selected from the assessor pool of the Leibniz-LSB@TUM. Assessors underwent regular weekly training sessions that focused on learning and applying a standardized sensory vocabulary. Training included evaluating individual reference odorant solutions and their mixtures, enabling assessors to consistently recognize and describe odor attributes. These sessions also served to identify potential individual olfactory limitations, including specific anosmias or hyposmias. The sensory assessments were carried out in individual booths located within a dedicated sensory analysis room maintained at 22 ± 1 °C.

For the quantitative olfactory profile analyses, aliquots of beer (15 mL) were transferred into lidded cylindrical ground-neck glass vessels (7 cm height, 3.5 cm i.d; VWR). Odor attributes were assessed orthonasally according to a predefined set of odor descriptors. Intensity scores were assigned on a scale ranging from 0 (not detectable) to 3 (strong), with increments of 0.1. Each descriptor was represented by a reference odorant dissolved in water at approximately one hundred times its orthonasal odor threshold concentration (OTC). The eight selected odor attributes and their corresponding reference compounds were “malty” (3-methylbutan-1-ol), “fruity” (ethyl hexanoate), “floral, honey” (2-phenylethan-1-ol), “caramel” (4-hydroxy-2,5-dimethylfuran-3(2*H*)-one), “clove” (2-methoxy-4-vinylphenol), “sweaty” (3-methylbutanoic acid), “cooked apple” ((*E*)-β-damascenone), and “banana” (3-methylbutyl acetate). For data evaluation, the ratings from all assessors were pooled and expressed as arithmetic mean values.

For the spiking experiment, the beer brewed with Km4 was spiked with an amount of 3-methylbutyl acetate to achieve a final concentration equal to the concentration previously determined in the beer brewed with CSa1. The quantitative olfactory profile of the spiked beer was orthonasally evaluated as described above.

## 3. Results and Discussion

### 3.1. Odor-Active Compounds in the Beers

GC–O, in combination with a comparative AEDA, applied to the volatile isolates obtained from the two non-alcoholic beers brewed under equal conditions with either Km4 or CSa1, resulted in 31 odorants. Of these 31 compounds, 27 were detected in both beer samples. The FD factors of the odorants ranged between 1 and 8192 (Table 1). Preliminary structural assignments were achieved by comparing RIs on two columns with different polarity (DB-FFAP and DB-5) and odor descriptions with published data compiled in the Leibniz-LSB@TUM Odorant Database [38]. The structure proposals were confirmed by analyzing the corresponding authentic reference compounds by GC–O and GC–MS in parallel with the beer volatile isolates.

The structures of 30 odorants were assigned. Despite all efforts, compound **28** remained unknown. Caramel-like smelling 4-hydroxy-2,5-dimethylfuran-3(2*H*)-one (HDMF; **22**) was identified as the compound with the highest FD factor of 8192 in the beer brewed with Km4. The FD factor in the beer brewed with CSa1 was only slightly lower, at 4096. The following four compounds had high FD factors in both beer samples, with the former value corresponding to the FD factor in the Km4 beer: 2-methoxy-4-vinylphenol (**26**; smoky, clove-like; FD factors 2048 and 4096), 2-phenylethan-1-ol (**20**; floral, honey-like; FD factors 4096 and 1024), phenylacetic acid (**30**; honey-like, beeswax-like; FD factors 1024 and 4096), and sotolon (**25**; fenugreek-like; FD factors 1024 and 512). All these compounds are already known as beer odorants, e.g., in wheat beer [39]. The results of the odorant identification are summarized in Table 1. In our previous paper on beer brewed with *T. delbrueckii*, all these odorants had already been detected via activity-guided odorant screening [18]. However, as far as we know, in non-alcoholic beers brewed with either Km4 or CSa1, these volatile compounds, except (*E*)-β-damascenone (**17**) [17], had not yet been characterized as important odorants using GC–O approaches.

About two-thirds of the compounds compiled in Table 1 showed no substantial FD factor differences between the two non-alcoholic beers. FD factors were either identical, such as for (*E*)-β-damascenone (**17**; both 512), or differed only by a factor of two, which cannot be interpreted as clear evidence of a quantitative difference, e.g., for HDMF (**22**; 8192 vs. 4096). Among the compounds with substantial FD factor differences between the beer brewed with Km4 and the beer brewed with CSa1, 3-methylbutyl acetate (**7**) showed the biggest difference. For this banana-like smelling odorant, an FD factor of 512 was obtained in the CSa1 beer, whereas it was not perceivable in the Km4 beer. For the compounds ethyl 2-methylbutanoate (**4**; fruity; FD factors 8 and 2), butanoic acid (**14**; sweaty; FD factors 32 and 8), hexanoic acid (**18**; sweaty; FD factors 4 and 1), 2,3-dihydromaltol (**19**; caramel-like; FD factors 16 and 4), octanoic acid (**23**; musty; FD factors 4 and <1), (6*Z*)-dodec-6-eno-γ-lactone (**29**; peach-like; FD factors 8 and 1), and phenylacetic acid (**30**; honey-like, beeswax-like; FD factors 4096 and 1024), the respective FD factors of the two beers also differed but to a lesser extent. Again, the beer brewed with CSa1 had a higher value in each case. On the other hand, odorants with substantially higher FD factors in the beer brewed with Km4 were 2-methylpropanoic acid (**13**; cheesy, sweaty; FD factors 64 and 8), methionol (**16**; cooked potato-like; FD factors 128 and 16), and 2-phenylethan-1-ol (**20**; floral, honey-like; FD factors 4096 and 1024).

The odorant screening in the non-alcoholic beers was further expanded to include highly volatile compounds. Substances with boiling points lower than that of the extraction solvent are not adequately captured by the solvent extraction/SAFE methodology, as they may be lost during the concentration step. To ensure that potentially relevant odorants were not missed, a comparative dilution analysis of static headspace samples was performed. This complementary approach enabled the assessment of highly volatile odorants. In headspace volumes of 20 mL, seven further odorants were detected (Table 2), six of which were perceivable in both beer samples. The FD factors ranged from 1 to 2 in the beer brewed with Km4 and from 1 to 4 in the beer brewed with CSa1. Structure assignments were based on the comparison of the RI on the DB-5 column, the odor quality perceived at the sniffing port during HS–GC–O, and the mass spectrum obtained by HS–GC–MS with data of authentic reference substances analyzed under the same conditions. This approach allowed for the structural assignment of all seven odorants. In the order of decreasing FD factors, these highly volatile odorants were malty smelling 2-methylpropanal (**34**) with FD factors of 2 in the Km4 beer and 4 in the CSa1 beer, dimethyl sulfide (**32**; cooked asparagus-like; FD factors 2 and 2), butane-2,3-dione (**35**; butter-like; FD factors 2 and 2), 3-methylbutanal (**37**; malty; FD factors 2 and 2), 2-methylbutanal (**38**; malty; FD factors 2 and 1), propanal (**33**; malty; FD factors 1 and 1), and ethyl acetate (**36**; solvent-like; FD factors <1 and 1). The FD factors were very similar; none of the highly volatile compounds showed a substantial difference between the two beer samples.

In summary, all identified compounds were previously reported in an alcoholic beer brewed with *S. cerevisiae* [18], indicating that there was no major qualitative difference in the odorants between alcoholic and non-alcoholic beers. Furthermore, the screening experiments revealed an almost identical set of odor-active compounds in the two beers brewed with Km4 and CSa1, respectively. However, the FD factors suggested quantitative differences in the key odorants between the two beers. Therefore, quantitation of the odorants was the essential next step [34].

### 3.2. Odorant Concentrations and OAVs

Thirty-four odorants previously detected during the odorant screening (cf. Table 1 and Table 2) were selected for quantitation in the two non-alcoholic beers brewed with either Km4 or CSa1. Quantitative determination of these odor-active compounds was carried out using GC–MS, GC×GC–MS, or HS–GC–MS, employing stable isotopically substituted odorants as internal standards. The combined concentration of 2- and 3-methylbutan-1-ol (**8a** and **b**) was initially measured by GC×GC–MS with (^2^H_2_)-3-methylbutan-1-ol serving as the internal standard. In a subsequent analytical step, the relative proportions of the two alcohols were determined following chromatographic separation on a nonpolar thick-film column using the GC–GC–MS system. Acetaldehyde (**39**), as a well-known beer odorant, was additionally quantitated by means of a commercial enzymatic assay.

The results of the odorant quantitations revealed concentrations between 0.02 µg/kg and 48,100 µg/kg, thus spanning over six orders of magnitude (Table 3). Seven compounds showed concentrations above 1 mg/kg in the Km4 and CSa1 beer, namely acetic acid (**11**; 48,100 µg/kg and 24,500 µg/kg), 3-methylbutan-1-ol (**8b**; 16,300 µg/kg and 7410 µg/kg), ethyl acetate (**36**; 6430 µg/kg and 3260 µg/kg), 2-methylbutan-1-ol (**8a**; 5330 µg/kg and 4290 µg/kg), acetaldehyde (**39**; 3450 µg/kg and 2140 µg/kg), 2-phenylethan-1-ol (**20**; 2240 µg/kg and 1660 µg/kg), and maltol (**21**; 1760 µg/kg and 1600 µg/kg). In addition, 3-methylbutyl acetate (**7**) also exceeded 1 mg/kg, albeit only in the CSa1 beer (5960 µg/kg).

To better assess the odor potency of individual compounds than with the FD factors, OAVs were calculated from the experimentally determined concentrations and the water-based OTCs (Table 3). In the Km4 beer, 21 out of the 35 previously quantitated odorants showed OAVs ≥ 1, and in the CSa1 beer, 22 odorants had an OAV ≥ 1. Among the odorants with OAVs ≥ 1, banana-like smelling 3-methylbutyl acetate (**7**) showed the highest OAV, namely 830, in the beer brewed with CSa1. Two additional odorants showed OAVs ≥100 in this beer: (*E*)-β-damascenone (**17**; OAV 280) and acetaldehyde (**39**; OAV 130). These two compounds were the only ones with OAVs ≥ 100 in the beer brewed with Km4, 160 and 220, respectively. Both were previously reported in an alcoholic beer brewed with *S. cerevisiae*, and OAVs were quite similar to those reported here in the non-alcoholic beers: 280 for acetaldehyde and 210 for (*E*)-β-damascenone [18]. The OAV range of <100 but >30 included 3-methylbutan-1-ol (**8b**) with an OAV of 74 in the Km4 beer and 34 in the CSa1 beer. In addition, methional (**12**) had an OAV of 62 in the Km4 beer.

Eighty percent of the quantitated compounds compiled in Table 3 showed only OAV factor differences of less than two-fold between the beer brewed with Km4 and the beer brewed with CSa1. OAV differences were particularly small for, e.g., ethyl 2-methylpropanoate (**2**; OAVs 24 vs. 18), 2-methylbutan-1-ol (**8a**; OAVs 4.4 vs. 3.6), HDMF (**22**; OAVs 9.6 vs. 9.0), 2-methoxy-4-vinylphenol (**26**; OAVs 3.3 vs. 4.4), 2-aminoacetophenone (**27**; OAVs 14 vs. 19), 3-methylbutanal (**37**; OAVs 9.2 vs. 10), and 2-methylbutanal (**38**; OAVs 2.3 vs. 2.5). By contrast, a clearly higher OAV was determined for 3-methylbutyl acetate (**7**) in the CSa1 beer than in the Km4 beer (830 vs. 8.2). This was the greatest OAV difference between the two beers for any of the quantitated compounds and corresponded well to the results of the comparative AEDA.

### 3.3. Sensory Characteristics of the Beers

The quantitative olfactory profiles of both non-alcoholic beers brewed with either Km4 or CSa1 showed patterns fairly characteristic of top-fermented beers, with high ratings for malty, fruity, and floral, honey-like aroma notes (Table 4). In particular, the Km4 beer had a quantitative olfactory profile similar to that of the ethanol-containing beer brewed with *S. cerevisiae* reported in our previous paper [18]. There were only minor differences in the floral, honey-like and clove-like aroma notes. These results suggested that *K. marxianus* (strain TUM G4KM) was a suitable non-*Saccharomyces* yeast for producing non-alcoholic beers with a balanced aroma and no off-flavors when used under the brewing conditions described in Section 2.1.

Apparent differences between the two non-alcoholic beers were the dominant banana note and the stronger fruitiness of the CSa1 beer. We hypothesized that 3-methylbutyl acetate (**7**), the compound that showed the greatest differences in FD factors, concentrations, and OAVs between the two beers, was responsible for the aroma difference in the two beers. To substantiate this hypothesis and demonstrate the crucial role of 3-methylbutyl acetate, a spiking experiment was performed. 3-Methylbutyl acetate was added to the Km4 beer in an amount corresponding to the concentration difference between the two beer samples. The spiked sample was orthonasally compared to the Km4 beer and the CSa1 beer. The intensities of the aroma notes were rated on a scale from 0 (not detectable) to 3 (strong). As a result, for the banana note, we obtained an average value of 2.1 for the spiked beer, which was close to the value of 2.2 obtained for the CSa1 beer and clearly higher than the value of 0.7 obtained for the Km4 beer (Table 4). Statistical comparison by means of two-tailed paired *t*-tests showed no significant difference between the spiked and the CSa1 beer (*p*-value = 0.8). In contrast, the comparison between the spiked and the Km4 beer revealed a very highly significant difference, as reflected by a *p*-value of 0.000006. Furthermore, for the fruity note, we obtained an average value of 1.9 for the spiked beer, which was close to the value of 2.0 obtained for the CSa1 beer and clearly higher than the value of 1.4 obtained for the Km4 beer. Using two-tailed paired *t*-tests, a *p*-value of 0.6 indicated no significant difference between the spiked and the CSa1 beer, whereas a *p*-value of 0.005 indicated a highly significant difference between the spiked and the Km4 beer. The results thus confirmed our hypothesis and finally revealed 3-methylbutyl acetate as the key odorant responsible for the intense banana note that differentiated the aroma of the CSa1 beer from the aroma of the Km4 beer. During the brewing process, *C. saturnus* (strain YHMH22AA-3H1) produced clearly more 3-methylbutyl acetate than *K. marxianus* (strain TUM G4KM). It is known that 3-methylbutyl acetate is formed by yeast when the amino acid L-leucine is broken down into 3-methylbutan-1-ol via the Ehrlich pathway, followed by esterification with acetyl-CoA. Trinh et al. already showed that *C. saturnus* enhanced the formation of 3-methylbutan-1-ol and its ester, 3-methylbutyl acetate, upon the addition of L-leucine [40].

## 4. Conclusions

In summary, using *K. marxianus* (strain TUM G4KM) or *C. saturnus* (strain YHMH22AA-3H1) for beer brewing resulted in non-alcoholic beers, both with well-balanced aromas. The CSa1 beer exhibited an intense banana note caused by a high concentration of 3-methylbutyl acetate. In the future, targeted quantitation of 3-methylbutyl acetate in CSa1 beers can be used to optimize aroma by adjusting fermentation parameters and thus contribute to the aroma diversification of beer.

## Figures and Tables

**Table 1:** Odorants in the Volatile Isolates Obtained from the Beer Brewed with *K. marxianus* (Km4) and the Beer Brewed with *C. saturnus* (CSa1).

No.	Odorant ^1^	Odor ^2^	RI ^3^		FD Factor ^4^	
			FFAP	DB-5	Km4 Beer	CSa1 Beer
**1**	ethyl propanoate	fruity	955	719	1	1
**2**	ethyl 2-methylpropanoate	fruity	981	772	4	4
**3**	ethyl butanoate	fruity	1033	806	2	1
**4**	ethyl 2-methylbutanoate	fruity	1050	854	2	8
**5**	ethyl 3-methylbutanoate	fruity	1059	853	<1	1
**6**	2-methylpropan-1-ol	malty	1094	639	2	4
**7**	3-methylbutyl acetate	banana	1123	881	<1	512
**8**	2-/3-methylbutan-1-ol ^5^	malty	1206	753	512	256
**9**	ethyl hexanoate	fruity	1249	998	<1	1
**10**	2-acetyl-1-pyrroline	roasty, popcorn	1335	926	64	32
**11**	acetic acid	vinegar	1440	656	256	128
**12**	methional	cooked potato	1451	907	32	64
**13**	2-methylpropanoic acid	cheesy, sweaty	1563	792	64	8
**14**	butanoic acid	sweaty	1623	830	8	32
**15**	2-/3-methylbutanoic acid ^5^	sweaty	1681	867	128	256
**16**	methionol	cooked potato	1720	983	128	16
**17**	(*E*)-β-damascenone	cooked apple	1821	1380	512	512
**18**	hexanoic acid	sweaty	1860	1016	1	4
**19**	2,3-dihydromaltol	caramel	1868	1079	4	16
**20**	2-phenylethan-1-ol	floral, honey	1916	1119	4096	1024
**21**	maltol	caramel	1978	1109	32	32
**22**	HDMF ^ 6^	caramel	2037	1083	8192	4096
**23**	octanoic acid	musty	2055	1265	<1	4
**24**	4-methylphenol	phenolic, horse stable	2093	1085	64	32
**25**	sotolon	fenugreek	2206	1104	1024	512
**26**	2-methoxy-4-vinylphenol	smoky, clove	2206	1308	2048	4096
**27**	2-aminoacetophenone	foxy	2234	1300	256	256
**28**	unknown	phenolic	2352	1212	4	4
**29**	(6*Z*)-dodec-6-eno-γ-lactone	peach	2406	1657	1	8
**30**	phenylacetic acid	honey, beeswax	2562	1265	1024	4096
**31**	vanillin	vanilla	2588	1498	64	32

^1^ Odorants showing an FD factor of ≥1 in at least one of the two samples; structure assignments were based on the comparison of the retention indices on two columns of different polarity (DB-FFAP, DB-5), the odor quality perceived at the sniffing port during GC–O, and the mass spectra obtained by GC–MS (CI and EI) with data of authentic reference substances analyzed under the same conditions. ^2^ Odor quality as perceived at the sniffing port during GC–O. ^3^ Retention index: calculated from the retention time of the odorant and the retention times of adjacent *n*-alkanes by linear interpolation. ^4^ Flavor dilution factor: dilution factor of the highest diluted sample obtained from the concentrated volatile isolates by serial dilution in which the odorant was detected by any of three assessors during GC–O. ^5^ The compounds were not separated on the DB-FFAP column used for AEDA; GC–MS(EI) indicated a mixture of both compounds. ^6^ HDMF: 4-hydroxy-2,5-dimethylfuran-3(2*H*)-one.

**Table 2:** Additional Odorants Detected in the Headspace above the Beer Brewed with *K. marxianus* (Km4) and the Beer Brewed with *C. saturnus* (CSa1).

No.	Odorant ^1^	Odor ^2^	RI ^3^ DB-5	FD Factor ^4^	
				Km4 Beer	CSa1 Beer
**32**	dimethyl sulfide	cooked asparagus	513	2	2
**33**	propanal	malty	537	1	1
**34**	2-methylpropanal	malty	568	2	4
**35**	butane-2,3-dione	butter	610	2	2
**36**	ethyl acetate	solvent	626	<1	1
**37**	3-methylbutanal	malty	658	2	2
**38**	2-methylbutanal	malty	663	2	1

^1^ Odorants showing an FD factor of ≥1 in at least one of the two samples; structure assignments were based on the comparison of the retention index on the DB-5 column, the odor quality perceived at the sniffing port during GC–O, and the mass spectra obtained by GC–MS (CI and EI) with data of authentic reference substances analyzed under the same conditions. ^2^ Odor quality as perceived at the sniffing port during GC–O. ^3^ Retention index: calculated from the retention time of the odorant and the retention times of adjacent *n*-alkanes by linear interpolation. ^4^ Flavor dilution factor: calculated as the ratio of the initial injection volume (20 mL) to the lowest injection volume in which the odorant was detected by any of three assessors during GC–O.

**Table 3:** Concentrations and OAVs of Important Odorants in the Beer Brewed with *K. marxianus* (Km4) and the Beer Brewed with *C. saturnus* (CSa1).

No. ^1^	Odorant	OTC ^2^ (µg/kg)	Km4 Beer		CSa1 Beer	
			Conc. ^3^ (µg/kg)	OAV ^4^	Conc. ^3^ (µg/kg)	OAV ^4^
**1**	ethyl propanoate	12	128	11	27.2	2.3
**2**	ethyl 2-methylpropanoate	0.089	2.13	24	1.62	18
**3**	ethyl butanoate	0.75	0.874	1.2	10.1	13
**4**	ethyl 2-methylbutanoate	0.13	≤0.02	<1	≤0.02	<1
**5**	ethyl 3-methylbutanoate	0.023	≤0.03	≤1	≤0.03	≤1
**7**	3-methylbutyl acetate	7.2	58.8	8.2	5960	830
**8a**	2-methylbutan-1-ol	1200	5330	4.4	4290	3.6
**8b**	3-methylbutan-1-ol	220	16,300	74	7410	34
**9**	ethyl hexanoate	1.2	0.528	<1	1.39	1.2
**10**	2-acetyl-1-pyrroline	0.053	≤0.03	<1	≤0.03	<1
**11**	acetic acid	5600	48,100	8.6	24,500	4.4
**12**	methional	0.43	26.8	62	7.46	17
**13**	2-methylpropanoic acid	60,000	602	<1	784	<1
**14**	butanoic acid	2400	259	<1	644	<1
**15**	2-/3-methylbutanoic acid ^5^	490	509	1.0	429	<1
**16**	methionol	36	689	19	235	6.5
**17**	(*E*)-β-damascenone	0.0060	0.937	160	1.69	280
**18**	hexanoic acid	4800	90.5	<1	273	<1
**19**	2,3-dihydromaltol	55	32.4	<1	63.6	1.2
**20**	2-phenylethan-1-ol	140	2240	16	1660	12
**21**	maltol	5000	1760	<1	1600	<1
**22**	HDMF ^ 6^	87	832	9.6	783	9.0
**24**	4-methylphenol	3.9	3.67	<1	3.48	<1
**25**	sotolon	1.7	1.19	<1	1.15	<1
**26**	2-methoxy-4-vinylphenol	7.6	24.9	3.3	33.8	4.4
**27**	2-aminoacetophenone	0.27	3.83	14	5.10	19
**30**	phenylacetic acid	68	415	6.1	620	9.1
**31**	vanillin	53	8.10	<1	10.2	<1
**33**	propanal	9.3	9.11	<1	8.04	<1
**34**	2-methylpropanal	0.49	6.91	14	3.83	7.8
**35**	butane-2,3-dione	0.96	4.03	4.2	6.61	6.9
**36**	ethyl acetate	12,000	6430	<1	3260	<1
**37**	3-methylbutanal	0.50	4.59	9.2	4.98	10
**38**	2-methylbutanal	1.5	3.45	2.3	3.71	2.5
**39**	acetaldehyde	16	3450	220	2140	130

^1^ Numbering according to Table 1 and Table 2. ^2^ Odor threshold concentration in water. Data were taken from our previous paper [18]. ^3^ Mean of triplicates: individual values and standard deviations are available in the Supplementary Materials File, Tables S2 and S3. ^4^ Odor activity value: calculated as the ratio of the concentration in the beer to the OTC. ^5^ Concentrations are sums of 2- and 3-methylbutanoic acid; OAVs were approximated by using the OTC of 3-methylbutanoic acid (490 μg/kg; the OTC of 2-methylbutanoic acid is 3100 µg/kg). ^6^ HDMF: 4-hydroxy-2,5-dimethylfuran-3(2*H*)-one.

**Table 4:** Intensity Ratings of Selected Odor Qualities in the Standard Beer Brewed with *S. cerevisiae* (Sc177), the Non-alcoholic Beer Brewed with *K. marxianus* (Km4), the Non-alcoholic Beer Brewed with *C. saturnus* (CSa1), and the Non-alcoholic Beer Brewed with *K. marxianus* and Spiked with 3-Methylbutyl Acetate.

Odor Quality	Intensity ^1^ in			
	Sc177 Beer ^2^	Km4 Beer	CSa1 Beer	Spiked Beer
malty	2.2	2.0	1.7	1.8
fruity	1.6	1.4	2.0	1.9
floral, honey	2.0	1.6	1.7	1.7
caramel	1.1	1.0	0.9	0.8
clove	0.4	0.7	0.5	0.6
sweaty	0.9	0.7	0.8	0.8
cooked apple	1.0	1.2	1.2	1.1
banana	0.8	0.7	2.2	2.1

^1^ Assessors rated the intensity of each descriptor on a scale from 0 to 3 with 0.1 increments and 0 = not detectable, 1 = weak, 2 = moderate, and 3 = strong. ^2^ Beer brewed with *S. cerevisiae* TUM 177 (5.2% ALC/VOL). Data were taken from our previous paper [18].

## Data Availability

The original contributions presented in this study are included in the article/Supplementary Materials. Further inquiries can be directed to the corresponding author.

## Supplementary Materials

The following supporting information can be downloaded at https://www.mdpi.com/article/10.3390/foods15173029/s1. Additional information on GC instruments; Table S1: Internal standards, quantifier ions, and calibration lines used in the quantitation assays; Table S2: Odorant concentrations in the beer brewed with *Kluyveromyces marxianus*; Table S3: Odorant concentrations in the beer brewed with *Cyberlindnera saturnus*.

## Abbreviations and Nomenclature

The following abbreviations are used in this manuscript:

2-acetyl-1-pyrroline	1-(3,4-dihydro-2*H*-pyrrol-5-yl)ethan-1-one
AEDA	aroma extract dilution analysis
2-aminoacetophenone	1-(2-aminophenyl)ethanone
CI	chemical ionization
(*E*)-β-damascenone	(2*E*)-1-(2,6,6-trimethylcyclohexa-1,3-dien-1-yl)but-2-en-1-one
2,3-dihydromaltol	5-hydroxy-6-methyl-2,3-dihydro-4*H*-pyran-4-one
dimethyl sulfide	(methylsulfanyl)methane
EI	electron ionization
FD factor	flavor dilution factor
FFAP	free fatty acid phase
FID	flame ionization detector
GC–MS	gas chromatography–mass spectrometry
GC–O	gas chromatography–olfactometry
HDMF	4-hydroxy-2,5-dimethylfuran-3(2*H*)-one
HS	headspace
maltol	3-hydroxy-2-methyl-4*H*-pyran-4-one
methional	3-(methylsulfanyl)propanal
methionol	3-(methylsulfanyl)propan-1-ol
OAV	odor activity value
OTC	odor threshold concentration
RI	retention index
SAFE	solvent-assisted flavor evaporation
sotolon	3-hydroxy-4,5-dimethylfuran-2(5*H*)-one
vanillin	4-hydroxy-3-methoxybenzaldehyde

## References

[1] Statista Research Department Pro-Kopf-Absatz von alkoholfreiem Bier in Deutschland in den Jahren 2014 bis 2024 mit einer Prognose bis 2027. https://de.statista.com/statistik/daten/studie/1364658/umfrage/pro-kopf-absatz-von-alkoholfreiem-bier-in-deutschland/.

[2] Brányik T., Silva D.P., Baszczyňski M., Lehnert R., Almeida e Silva J.B. (2012). A review of methods of low alcohol and alcohol-free beer production. J. Food Eng..

[3] Bellut K., Arendt E.K. (2019). Chance and challenge: Non-*Saccharomyces* yeasts in nonalcoholic and low alcohol beer brewing—A review. J. Am. Soc. Brew. Chem..

[4] Piornos J.A., Koussissi E., Balagiannis D.P., Brouwer E., Parker J.K. (2022). Alcohol-free and low-alcohol beers: Aroma chemistry and sensory characteristics. Compr. Rev. Food Sci. Food Saf..

[5] Methner Y., Hutzler M., Zarnkow M., Prowald A., Endres F., Jacob F. (2022). Investigation of non-*Saccharomyces* yeast strains for their suitability for the production of non-alcoholic beers with novel flavor profiles. J. Am. Soc. Brew. Chem..

[6] Krogerus K., Eerikainen R., Aisala H., Gibson B. (2022). Repurposing brewery contaminant yeast as production strains for low-alcohol beer fermentation. Yeast.

[7] Postigo V., Sánchez A., Cabellos J.M., Arroyo T. (2022). New approaches for the fermentation of beer: Non-*Saccharomyces* yeasts from wine. Fermentation.

[8] Yabaci Karaoglan S., Jung R., Gauthier M., Kinčl T., Dostálek P. (2022). Maltose-negative yeast in non-alcoholic and low-alcoholic beer production. Fermentation.

[9] Qin X., Li F., Bo W., Li W., Dong X. (2025). The non-*Saccharomyces* yeasts as novel starter cultures to innovate beer brewing. CyTA–J. Food.

[10] Canonico L., Comitini F., Agarbati A., Ciani M. (2026). A comprehensive review of non-conventional yeasts: Innovation in craft beer production. Foods.

[11] Gamero A., Quintilla R., Groenewald M., Alkema W., Boekhout T., Hazelwood L. (2016). High-throughput screening of a large collection of non-conventional yeasts reveals their potential for aroma formation in food fermentation. Food Microbiol..

[12] Ravasio D., Carlin S., Boekhout T., Groenewald M., Vrhovsek U., Walther A., Wendland J. (2018). Adding flavor to beverages with non-conventional yeasts. Fermentation.

[13] Methner Y., Hutzler M., Matoulková D., Jacob F., Michel M. (2019). Screening for the brewing ability of different non-*Saccharomyces* yeasts. Fermentation.

[14] Canonico L., Zannini E., Ciani M., Comitini F. (2021). Assessment of non-conventional yeasts with potential probiotic for protein-fortified craft beer production. LWT Food Sci. Technol..

[15] Vaštík P., Brunner J., Jung R., Klempová T., Furdíková K., Šmogrovičová D., Dostálek P. (2025). Functional non-alcoholic beer fermented with potential probiotic yeasts. Beverages.

[16] Vaštík P., Rosenbergova Z., Furdikova K., Klempova T., Sismis M., Smogrovicova D. (2022). Potential of non-*Saccharomyces* yeast to produce non-alcoholic beer. FEMS Yeast Res..

[17] Methner Y., Dancker P., Maier R., Latorre M., Hutzler M., Zarnkow M., Steinhaus M., Libkind D., Frank S., Jacob F. (2022). Influence of varying fermentation parameters of the yeast strain *Cyberlindnera saturnus* on the concentrations of selected flavor components in non-alcoholic beer focusing on (*E*)-*β*-damascenone. Foods.

[18] Frank S., Maier R., Steinhaus M. (2025). Characterization of the major odorants in beer brewed with *Torulaspora delbrueckii*—Differences to beer brewed with *Saccharomyces cerevisiae*. ACS Food Sci. Technol..

[19] Central European Commission for Brewing Analysis MEBAK Methods Database. https://www.mebak.org/methoden-datenbank.

[20] Kiefl J., Pollner G., Schieberle P. (2013). Sensomics analysis of key hazelnut odorants (*Corylus avellana* L. ‘Tonda Gentile’) using comprehensive two-dimensional gas chromatography in combination with time-of-flight mass spectrometry (GCxGC-TOF-MS). J. Agric. Food Chem..

[21] Fuhrmann E. (1998). Untersuchung über das Aroma von Äpfeln in Abhängigkeit von der Sorte und über die Beziehung zwischen Struktur und Geruch daran beteiligter Ester. PhD Thesis.

[22] Neiens S.D., Steinhaus M. (2018). Odor-active compounds in the special flavor hops Huell Melon and Polaris. J. Agric. Food Chem..

[23] Steinhaus M., Sinuco D., Polster J., Osorio C., Schieberle P. (2009). Characterization of the key aroma compounds in pink guava (*Psidium guajava* L.) by means of aroma re-engineering experiments and omission tests. J. Agric. Food Chem..

[24] Grimm J.E., Steinhaus M. (2019). Characterization of the major odor-active compounds in jackfruit pulp. J. Agric. Food Chem..

[25] Steinhaus M., Schieberle P. (2005). Characterization of odorants causing an atypical aroma in white pepper powder (*Piper nigrum* L.) based on quantitative measurements and orthonasal breakthrough thresholds. J. Agric. Food Chem..

[26] Sen A., Grosch W. (1991). Synthesis of six deuterated sulfur containing odorants to be used as internal standards in quantification assays. Eur. Food Res. Technol..

[27] Sen A., Laskawy G., Schieberle P., Grosch W. (1991). Quantitative determination of beta-damascenone in foods using a stable isotope dilution assay. Agric. Food Chem..

[28] Harada R., Iwasaki M. (1983). Syntheses of maltol and ethylmaltol. Agric. Biol. Chem..

[29] Blank I., Schieberle P., Grosch W., Schreier P., Winterhalter P. (1993). Quantification of the flavour compounds 3-hydroxy-4,5-dimethyl-2(5*H*)-furanone and 5-ethyl-3-hydroxy-4-methyl-2(5*H*)-furanone by stable isotope dilution assay. Progress in Flavour Precursor Studies.

[30] Semmelroch P., Laskawy G., Blank I., Grosch W. (1995). Determination of potent odourants in roasted coffee by stable isotope dilution assays. Flavour Fragr. J..

[31] Mall V., Schieberle P. (2017). Evaluation of key aroma compounds in processed prawns (whiteleg shrimp) by quantitation and aroma recombination experiments. J. Agric. Food Chem..

[32] Granvogl M., Beksan E., Schieberle P. (2012). New insights into the formation of aroma-active strecker aldehydes from 3-oxazolines as transient intermediates. J. Agric. Food Chem..

[33] Schieberle P., Hofmann T. (1997). Evaluation of the character impact odorants in fresh strawberry juice by quantitative measurements and sensory studies on model mixtures. J. Agric. Food Chem..

[34] Steinhaus M., Tranchida P. (2019). Gas chromatography-olfactometry: Principles, practical aspects and applications in food analysis. Advanced Gas Chromatography in Food Analysis.

[35] van Den Dool H., Kratz P.D. (1963). A generalization of the retention index system including linear temperature programmed gas-liquid partition chromatography. J. Chromatogr..

[36] Engel W., Bahr W., Schieberle P. (1999). Solvent assisted flavour evaporation—A new and versatile technique for the careful and direct isolation of aroma compounds from complex food matrices. Eur. Food Res. Technol..

[37] Bemelmanns J.M.H., Land G.G., Nursten H.E. (1979). Review of isolation and concentration techniques. Progress in Flavour Research.

[38] Kreissl J., Mall V., Steinhaus P., Steinhaus M. (2022). Leibniz-LSB@TUM Odorant Database.

[39] Langos D., Granvogl M., Schieberle P. (2013). Characterization of the key aroma compounds in two Bavarian wheat beers by means of the sensomics approach. J. Agric. Food Chem..

[40] Trinh T.-T.-T., Yu B., Curran P., Liu S.-Q. (2012). Formation of aroma compounds during longan juice fermentation by *Williopsis saturnus* var. *saturnus* with the addition of selected amino acids. J. Food Process. Preserv..

