# Supplementary material for: Characterization of the Major Odorants in Non-Alcoholic Beers Brewed with Either Kluyveromyces marxianus or Cyberlindnera saturnus
Source: Foods. 2026 Aug 27;15(17):3029. doi: 10.3390/foods15173029 (PMC13564787; doi:10.3390/foods15173029)
Supplement: Supplementary file 1 [file foods-15-03029-s001.zip › foods-4489251-supplementary.pdf]

# Supplementary Materials

## Characterization of the Major Odorants in Non-Alcoholic Beers Brewed with either *Kluyveromyces marxianus* or *Cyberlindnera saturnus*

Stephanie Frank, Robin Maier, and Martin Steinhaus*

Leibniz Institute for Food Systems Biology at the Technical University of Munich,
Lise-Meitner-Straße 34, 85354 Freising, Germany

*E-mail: m.steinhaus.leibniz-lsb@tum.de

**GC–O/FID.** A Trace GC Ultra gas chromatograph (Thermo Scientific; Dreieich, Germany) was equipped with a cold on-column injector, a flame ionization detector (FID), and a sniffing port tailor-made as detailed in *J. Agric. Food Chem.* **2008**, *56*, 4120–4127. The fused silica columns used were either a DB-FFAP, 30 m × 0.32 mm i.d., 0.25 µm film or a DB-5, 30 m × 0.25 mm i.d., 0.25 µm film (both Agilent Technologies; Waldbronn, Germany). The carrier gas was helium at 65 kPa (DB-FFAP) and 90 kPa (DB-5) constant pressure. The injection volume was 1 µL. The initial oven temperature was 40 °C for 2 min and then was ramped at 6 °C/min to 230 °C for DB-FFAP and to 240 °C for DB-5. The column effluent was divided into two parts (1:1) using a deactivated Y-shaped glass splitter in combination with deactivated fused silica capillaries (50 cm × 0.25 mm i.d.) connecting the splitter to the FID (250 °C) and the sniffing port (250 °C).

**HS–GC–O/MS.** A Trace GC Ultra gas chromatograph (Thermo) was equipped with a Combi PAL autosampler (CTC Analytics; Zwingen, Switzerland) and a cold on-column injector (Thermo). The gas chromatograph featured a Cold Trap 915 (Thermo) and a DB-5 column, 30 m × 0.25 mm i.d., 1.0 µm film (Agilent). The carrier gas was helium at 75 kPa constant pressure. The initial oven temperature was 0 °C for 2 min and then was ramped at 6 °C/min to 110 °C and finally was ramped at 40 °C/min to 230 °C. The column effluent was divided into three parts (1:1:2) using deactivated Y-shaped glass splitters in combination with deactivated fused silica capillaries (50 cm × 0.25 mm i.d.) connecting the splitters to the FID (250 °C), to the sniffing port (230 °C), and the Saturn 2100 ion trap mass spectrometer (Varian; Darmstadt, Germany). The mass spectrometer was operated in CI mode with methanol as the reagent gas. For data analysis, MS Workstation software (Agilent) was employed.

**GC–MS.** A gas chromatograph 7890B (Agilent) was equipped with a GC 80 autosampler (Agilent) and a multimode injector (Agilent). The fused silica column used was a DB-FFAP, 30 m × 0.25 mm i.d., 0.25 µm film (Agilent). The carrier gas was helium at 1.0 mL/min constant flow. The injection volume was 2 µL. The initial oven temperature was 40 °C for 2 min and then was ramped at 6 °C/min to 230 °C. The column was connected to a Saturn 240 ion trap mass spectrometer (Agilent). The mass spectrometer was operated in CI mode with methanol as the reagent gas. For data analysis, MS Workstation software (Agilent) was employed.

**GC×GC–MS.** A 6890 Plus gas chromatograph (Agilent) was equipped with a Combi PAL autosampler (CTC Analytics), a KAS4 injector (Gerstel; Mülheim/Ruhr, Germany), and a fused silica column DB-FFAP, 25 m × 0.25 mm i.d., 0.25 µm film (Agilent). The end of the column was connected to a second fused silica column, which was a DB-5, 2 m × 0.15 mm i.d., 0.30 µm film (Varian). At the beginning of the second column, a liquid nitrogen-cooled dual-stage quad-jet thermal modulator (Leco; Mönchengladbach, Germany) was used to collect the volatiles eluted from the first column in discrete portions (4 s), which then were rechromatographed on the major part of the second column. This part

was installed in a secondary oven located inside the primary oven. The end of this column was connected to a Pegasus III time-of-flight mass spectrometer (Leco). The mass spectrometer was operated in EI mode at 70 eV, with a scan range of *m*/*z* 35–300, and a scan rate of 100 spectra/s. The carrier gas was helium at 2.0 mL/min constant flow. The injection volume was 2 µL. The initial oven temperature in the first dimension was 40 °C for 2 min and then was ramped at 6 °C/min to 230 °C. The initial oven temperature in the second dimension was 70 °C for 2 min and then was ramped at 6 °C/min to 250 °C. For data analysis, ChromSpace software (Markes International; Llantrisant, UK) was employed.

**GC–GC–MS.** A Trace GC Ultra gas chromatograph (Thermo) was equipped with a cold on-column injector (Thermo). The fused silica columns used were a DB-FFAP, 30 m × 0.32 mm i.d., 0.25 µm film (Agilent) in the first dimension and a DB-5, 30 m × 0.25 mm i.d., 1.00 µm film (Agilent) in the second dimension. The first column end was connected to a moving column stream switching (MCSS) device (Thermo), conveying the eluate via deactivated fused silica capillaries time-programmed either simultaneously to an FID (250 °C) and a sniffing port (230 °C) or via a heated (250 °C) hose to a cold trap located in the oven of a CP 3800 gas chromatograph (Varian). The cold trap consisted of a piece of steel tubing housing the capillary and could be cooled by liquid nitrogen. The end of the capillary was connected to the second fused silica column. The end of this column was connected to a Saturn 2000 ion trap mass spectrometer (Varian). The mass spectrometer was operated in CI mode with methanol as the reagent gas. The carrier gas was helium at 100 kPa constant pressure, which was also used as a makeup gas for the MCSS device (50 kPa). The injection volume was 2 µL. The initial oven temperature in the first dimension was 40 °C for 2 min and then was ramped at 6 °C/min to 230 °C. The initial oven temperature in the second dimension was 35 °C for 2 min and then was ramped at 6 °C/min to 120 °C and finally was ramped at 30°C/min to 240 °C. For data analysis, MS Workstation software (Agilent) was employed.

**Table S1.** Internal Standards, Quantifier Ions, and Calibration Lines Used in the Quantitation Assays

| odorant | standard | quantifier ions (*m*/*z*) | | calibration line equation[a] | $R^2$ |
|---|---|---|---|---|---|
| | | analyte | standard | | |
| **1** | ($^{2}H_{3}$)-**1** | 103 | 106 | y = 0.9543x − 0.1268 | 0.998 |
| **2** | ($^{2}H_{5}$)-**2** | 117 | 122 | y = 0.9673x − 0.0361 | 0.998 |
| **3** | ($^{2}H_{3}$)-**3** | 88 | 91 | y = 0.8642x + 0.1442 | 0.999 |
| **4** | ($^{2}H_{3}$)-**5** | 131 | 134 | y = 1.0235x − 0.1102 | 0.998 |
| **5** | ($^{2}H_{3}$)-**5** | 131 | 134 | y = 1.0248x − 0.0473 | 1.000 |
| **7** | ($^{2}H_{11}$)-**7** | 87 | 91 | y = 0.8728x + 0.1101 | 0.998 |
| **8a/b** | ($^{2}H_{2}$)-**8b** | 70 | 72 | y = 0.8099x + 0.0884 | 0.998 |
| **9** | ($^{2}H_{10–11}$)-**9** | 99 | 109+110 | y = 0.7269x − 0.0370 | 0.999 |
| **10** | ($^{13}C_{5}$)-**10** | 112 | 117 | y = 0.9838x − 0.0814 | 0.997 |
| **11** | ($^{2}H_{3}$)-**11** | 75 | 78 | y = 0.8948x + 0.1165 | 0.999 |
| **12** | ($^{2}H_{3}$)-**12** | 104 | 107 | y = 0.9684x − 0.0230 | 1.000 |
| **13** | ($^{2}H_{7}$)-**13** | 103 | 110 | y = 0.9266x + 0.1731 | 0.997 |
| **14** | ($^{2}H_{2}$)-**14** | 89 | 91 | y = 1.1679x − 0.1642 | 0.999 |
| **15**[b] | ($^{2}H_{2}$)-**15** | 103 | 105 | y = 0.8662x + 0.0969 | 0.997 |
| **16** | ($^{2}H_{3}$)-**16** | 106 | 109 | y = 0.7823x + 0.1113 | 0.997 |
| **17** | ($^{2}H_{4–7}$)-**17** | 121 | 125–128 | y = 0.9610x − 0.1695 | 0.999 |
| **18** | ($^{2}H_{3}$)-**18** | 99 | 102 | y = 0.9138x + 0.0276 | 1.000 |
| **19** | ($^{13}C_{2}$)-**21** | 128 | 128 | y = 1.0613x − 0.1595 | 0.999 |
| **20** | ($^{2}H_{5}$)-**20** | 122 | 127 | y = 0.8556x + 0.0307 | 0.999 |
| **21** | ($^{13}C_{2}$)-**21** | 126 | 128 | y = 1.0100x + 0.1985 | 0.996 |
| **22** | ($^{13}C_{2}$)-**22** | 128 | 130 | y = 1.0376x − 0.0064 | 0.998 |
| **24** | ($^{2}H_{6–8}$)-**24** | 108 | 114–116 | y = 1.0422x + 0.0723 | 0.999 |
| **25** | ($^{13}C_{2}$)-**25** | 128 | 130 | y = 1.1123x + 0.1304 | 0.999 |
| **26** | ($^{2}H_{3}$)-**26** | 150 | 153 | y = 0.9623x + 0.0873 | 0.999 |
| **27** | ($^{2}H_{3}$)-**27** | 135 | 138 | y = 0.8657x − 0.0968 | 0.997 |
| **30** | ($^{13}C_{2}$)-**30** | 136 | 138 | y = 0.9930x + 0.0566 | 1.000 |
| **31** | ($^{2}H_{3}$)-**31** | 152 | 155 | y = 0.9760x + 0.0796 | 0.999 |
| **33** | ($^{2}H_{2}$)-**33** | 59 | 61 | y = 0.8842x + 0.0691 | 0.998 |
| **34** | ($^{2}H_{2}$)-**34** | 73 | 75 | y = 0.9334x − 0.0679 | 0.998 |
| **35** | ($^{13}C_{4}$)-**35** | 87 | 91 | y = 1.0921x − 0.0683 | 0.998 |
| **36** | ($^{13}C_{2}$)-**36** | 89 | 91 | y = 0.9897x + 0.0313 | 1.000 |
| **37** | ($^{2}H_{2}$)-**37** | 87 | 89 | y = 1.0264x − 0.1034 | 0.998 |
| **38** | ($^{2}H_{2}$)-**38** | 87 | 89 | y = 1.1997x − 0.1015 | 0.998 |

[a]y = peak area counts standard / peak area counts analyte; x = concentration standard (µg/mL) / concentration analyte (µg/mL). [b]2- and 3-methylbutanoic acid were quantitated as a sum, using ($^{2}H_{2}$)-3-methylbutanoic acid as the internal standard.

**Table S2.** Odorant Concentrations in the Beer Brewed with *Kluyveromyces marxianus*

| odorant | concentration (µg/kg) | | | |
|---|---|---|---|---|
| | exp. 1[a] | exp. 2[a] | exp. 3[a] | mean ± SD (CV)[b] |
| **1** | 133 | 120 | 131 | 128 ± 7 (5%) |
| **2** | 1.99 | 2.08 | 2.33 | 2.13 ± 0.18 (8%) |
| **3** | 0.817 | 0.912 | 0.892 | 0.874 ± 0.050 (6%) |
| **4** | ≤0.0119[c] | ≤0.0183[c] | ≤0.0146[c] | |
| **5** | ≤0.0247[c] | ≤0.0326[c] | ≤0.0268[c] | |
| **7** | 56.2 | 60.4 | 59.9 | 58.8 ± 2.3 (4%) |
| **8a** | 5010 | 5640 | 5350 | 5330 ± 320 (6%) |
| **8b** | 15900 | 16900 | 16200 | 16300 ± 510 (3%) |
| **9** | 0.560 | 0.515 | 0.510 | 0.528 ± 0.028 (5%) |
| **10** | ≤0.0254[c] | ≤0.0179[c] | ≤0.0138[c] | |
| **11** | 54600 | 43900 | 45600 | 48100 ± 5800 (12%) |
| **12** | 25.0 | 27.9 | 27.5 | 26.8 ± 1.6 (6%) |
| **13** | 663 | 546 | 598 | 602 ± 59 (10%) |
| **14** | 261 | 255 | 262 | 259 ± 4 (1%) |
| **15**[d] | 461 | 552 | 515 | 509 ± 46 (9%) |
| **16** | 676 | 672 | 720 | 689 ± 27 (4%) |
| **17** | 1.01 | 0.909 | 0.893 | 0.937 ± 0.063 (7%) |
| **18** | 92.2 | 95.8 | 83.5 | 90.5 ± 6.3 (7%) |
| **19** | 34.2 | 31.8 | 31.2 | 32.4 ± 1.6 (5%) |
| **20** | 2200 | 2300 | 2220 | 2240 ± 53 (2%) |
| **21** | 1630 | 1870 | 1780 | 1760 ± 120 (7%) |
| **22** | 782 | 837 | 877 | 832 ± 48 (6%) |
| **24** | 3.84 | 3.69 | 3.49 | 3.67 ± 0.17 (5%) |
| **25** | 1.10 | 1.25 | 1.21 | 1.19 ± 0.08 (6%) |
| **26** | 25.1 | 24.3 | 25.3 | 24.9 ± 0.5 (2%) |
| **27** | 4.18 | 3.59 | 3.71 | 3.83 ± 0.31 (8%) |
| **30** | 414 | 419 | 413 | 415 ± 3 (1%) |
| **31** | 7.96 | 8.08 | 8.26 | 8.10 ± 0.15 (2%) |
| **33** | 9.43 | 9.07 | 8.83 | 9.11 ± 0.30 (3%) |
| **34** | 7.34 | 6.52 | 6.88 | 6.91 ± 0.41 (6%) |
| **35** | 3.97 | 3.85 | 4.26 | 4.03 ± 0.21 (5%) |
| **36** | 6640 | 6270 | 6390 | 6430 ± 190 (3%) |
| **37** | 4.42 | 4.37 | 4.99 | 4.59 ± 0.34 (7%) |
| **38** | 3.13 | 3.63 | 3.58 | 3.45 ± 0.28 (8%) |
| **39** | 3030 | 3570 | 3740 | 3450 ± 370 (11%) |

[a]Individual concentrations were rounded to three significant digits for presentation. [b]Mean values were calculated from the unrounded concentrations of the individual experiments and finally rounded to three significant digits; SD, standard deviation; CV, coefficient of variation. [c]No analyte peak was observed; maximum values were derived from the integration of the background noise. [d]Sum of isomers.

**Table S3.** Odorant Concentrations in the Beer Brewed with *Cyberlindnera saturnus*

| odorant | concentration (µg/kg) | | | |
|---|---|---|---|---|
| | exp. 1[a] | exp. 2[a] | exp. 3[a] | mean ± SD (CV)[b] |
| **1** | 26.6 | 27.4 | 27.5 | 27.2 ± 0.5 (2%) |
| **2** | 1.53 | 1.66 | 1.67 | 1.62 ± 0.08 (5%) |
| **3** | 9.59 | 10.8 | 9.84 | 10.1 ± 0.7 (6%) |
| **4** | ≤0.0174[c] | ≤0.0127[c] | ≤0.0195[c] | |
| **5** | ≤0.0279[c] | ≤0.0314[c] | ≤0.0228[c] | |
| **7** | 6130 | 5840 | 5920 | 5960 ± 150 (2%) |
| **8a** | 4470 | 4060 | 4330 | 4290 ± 210 (5%) |
| **8b** | 7580 | 7210 | 7440 | 7410 ± 190 (3%) |
| **9** | 1.43 | 1.35 | 1.40 | 1.39 ± 0.04 (3%) |
| **10** | ≤0.0131[c] | ≤0.0192[c] | ≤0.0286[c] | |
| **11** | 25400 | 23900 | 24200 | 24500 ± 790 (3%) |
| **12** | 7.61 | 7.50 | 7.30 | 7.46 ± 0.15 (2%) |
| **13** | 842 | 732 | 779 | 784 ± 55 (7%) |
| **14** | 634 | 649 | 647 | 644 ± 7 (1%) |
| **15**[d] | 448 | 420 | 419 | 429 ± 16 (4%) |
| **16** | 243 | 230 | 234 | 235 ± 7 (3%) |
| **17** | 1.75 | 1.59 | 1.73 | 1.69 ± 0.09 (5%) |
| **18** | 264 | 275 | 281 | 273 ± 9 (3%) |
| **19** | 59.6 | 64.7 | 66.5 | 63.6 ± 3.6 (6%) |
| **20** | 1830 | 1700 | 1450 | 1660 ± 190 (12%) |
| **21** | 1660 | 1560 | 1590 | 1600 ± 51 (3%) |
| **22** | 789 | 744 | 816 | 783 ± 36 (5%) |
| **24** | 3.39 | 3.36 | 3.68 | 3.48 ± 0.17 (5%) |
| **25** | 1.20 | 1.08 | 1.17 | 1.15 ± 0.06 (5%) |
| **26** | 32.9 | 33.6 | 34.8 | 33.8 ± 1.0 (3%) |
| **27** | 4.93 | 4.72 | 5.66 | 5.10 ± 0.49 (10%) |
| **30** | 608 | 633 | 620 | 620 ± 13 (2%) |
| **31** | 10.5 | 10.1 | 10.1 | 10.2 ± 0.2 (2%) |
| **33** | 8.03 | 7.77 | 8.34 | 8.04 ± 0.29 (4%) |
| **34** | 3.71 | 3.78 | 4.01 | 3.83 ± 0.15 (4%) |
| **35** | 6.36 | 7.03 | 6.41 | 6.61 ± 0.37 (6%) |
| **36** | 3450 | 3230 | 3090 | 3260 ± 180 (6%) |
| **37** | 5.25 | 4.64 | 5.05 | 4.98 ± 0.31 (6%) |
| **38** | 3.60 | 3.86 | 3.67 | 3.71 ± 0.14 (4%) |
| **39** | 2320 | 1960 | 2140 | 2140 ± 180 (8%) |

[a]Individual concentrations were rounded to three significant digits for presentation. [b]Mean values were calculated from the unrounded concentrations of the individual experiments and finally rounded to three significant digits; SD, standard deviation; CV, coefficient of variation. [c]No analyte peak was observed; maximum values were derived from the integration of the background noise. [d]Sum of isomers.
